# MG-132 reduces virus release in Bovine herpesvirus-1 infection

**DOI:** 10.1038/s41598-017-13717-1

**Published:** 2017-10-17

**Authors:** Filomena Fiorito, Valentina Iovane, Antonietta Cantiello, Annarosaria Marullo, Luisa De Martino, Giuseppe Iovane

**Affiliations:** 1 Istituto Zooprofilattico Sperimentale del Mezzogiorno, Portici, 80055 Naples, Italy; 2 Department of Veterinary Medicine and Animal Production, University of Naples Federico II, 80137 Naples, Italy; 3 Department of Pharmacy, University of Salerno, Fisciano, Salerno, 84084 Italy; 4 Department of Veterinary Medical Sciences, Alma Mater Studiorum, University of Bologna, Bologna, 40126 Italy

## Abstract

Bovine herpesvirus 1 (BoHV-1) can provoke conjunctivitis, abortions and shipping fever. BoHV-1 infection can also cause immunosuppression and increased susceptibility to secondary bacterial infections, leading to pneumonia and occasionally to death. Herein, we investigated the influence of MG-132, a proteasome inhibitor, on BoHV-1 infection in bovine kidney (MDBK) cells. Infection of MDBK cells with BoHV-1 induces apoptotic cell death that enhances virus release. Whereas, MG-132 inhibited virus-induced apoptosis and stimulated autophagy. Protein expression of viral infected cell protein 0 (bICP0), which is constitutively expressed during infection and is able to stimulate Nuclear factor kappa B (NF-κB), was completely inhibited by MG-132. These results were accompanied by a significant delay in the NF-κB activation. Interestingly, the efficient virus release provoked by BoHV-1-induced apoptosis was significantly reduced by MG-132. Overall, this study suggests that MG-132, through the activation of autophagy, may limit BoHV-1 replication during productive infection, by providing an antiviral defense mechanism.

## Introduction

Bovine herpesvirus 1 (BoHV-1), a double-stranded DNA virus, is an important pathogen that in cattle can provoke infectious bovine rhinotracheitis (IBR), conjunctivitis, abortions and shipping fever, which is a complicated infection of the upper respiratory tract. BoHV-1 initiates the disorder through immunosuppression that could render the animals more susceptible to secondary bacterial infections, leading to pneumonia and occasionally to death^1,2^. BoHV-1 establishes latency in sensory neurons of the infected host. Reactivation from latency is stimulated by dexamethasone treatment or increases in natural corticosteroids resulting in virus shedding and spread to susceptible hosts^1,2^. The genes of BoHV-1, like other members of the alphaherpesvirus subfamily, are expressed in three temporally distinct phases identified as immediate-early (IE), early (E) and late (L) and it is generally approved that tissue-specific factors mediate pathogenesis and/or latency by influencing viral gene expression^1^. bICP0, the bovine homologue of herpes simplex virus type 1 (HSV-1) ICP0, regulates all three these phases by acting as a strong activator or as a repressor of specific viral promoters and is constitutively expressed during infection in permissive cells^1,3,4^. Thus, immediate-early bICP0 is considered to be the major regulatory protein that stimulates productive infection and inhibits interferon dependent transcription^5^. Infection of permissive cells (MDBK) with BoHV-1 leads to rapid cell death, partially due to apoptosis, which occurs during the late stages of infection by the activation of caspases, through modulation of Bcl-2 family members^4,6–12^. In the absence of viral gene expression, bICP0 indirectly induces caspase 3 activation and apoptosis^13^.

Found in almost all mammalian cell types, the transcription factor NF-κB regulates a wide range of genes important in development and prevention of apoptosis. Normally, NF-κB is sequestered within cytoplasm by virtue of its association with inhibitor IκBα. NF-κB can be released after that IκBα is phosphorylated and degraded, via the ubiquitin and proteasome pathway. As a result, the released NF-κB will be translocated into the nucleus, where it works as a transcriptional regulator of many related genes, playing critical role in inflammation, immunity, cell proliferation, differentiation, and survival^14,15^. Several viruses use cellular signaling pathways, like NF-κB, to stimulate viral gene expression^16^. Immediate early protein bICP0, could specifically stimulate NF-κB responsive reporter gene expression in different cell lines and induce NF-κB to translocate from cytoplasm into the nucleus where it promotes NF-κB DNA binding affinity^17^.

As above reported, viruses utilize various cellular signaling pathways during the course of their replication. The ubiquitin-proteasome system seems to be a cellular pathway that viruses use for their own benefit. Several studies showed that proteasome inhibitors can alter virus replication by playing significant roles in replication cycle. For example, proteasome inhibitors decreased immediate early and late proteins expression in HSV-1^18^, have a role in post-entry stages of HSV-1 infection^19–24^, and also facilitate the entry of HSV-1 at a post-penetration step^25^.

In this study, we used MG-132, a synthetic peptide inhibitor of the aldehyde proteasome pathway. Hence, MDBK cells, epithelial-like bovine cells commonly used for growing and assaying BoHV-1, were infected with BoHV-1 virus (Cooper strain), in the presence or absence of MG-132, and we examined the pathway of BoHV-1-induced apoptosis, viral and cellular proteins, and analyzed virus replication in infected cells.

## Results

### MG-132 decreases cytotoxicity of MDBK during BoHV-1 infection

**Figure 1:**
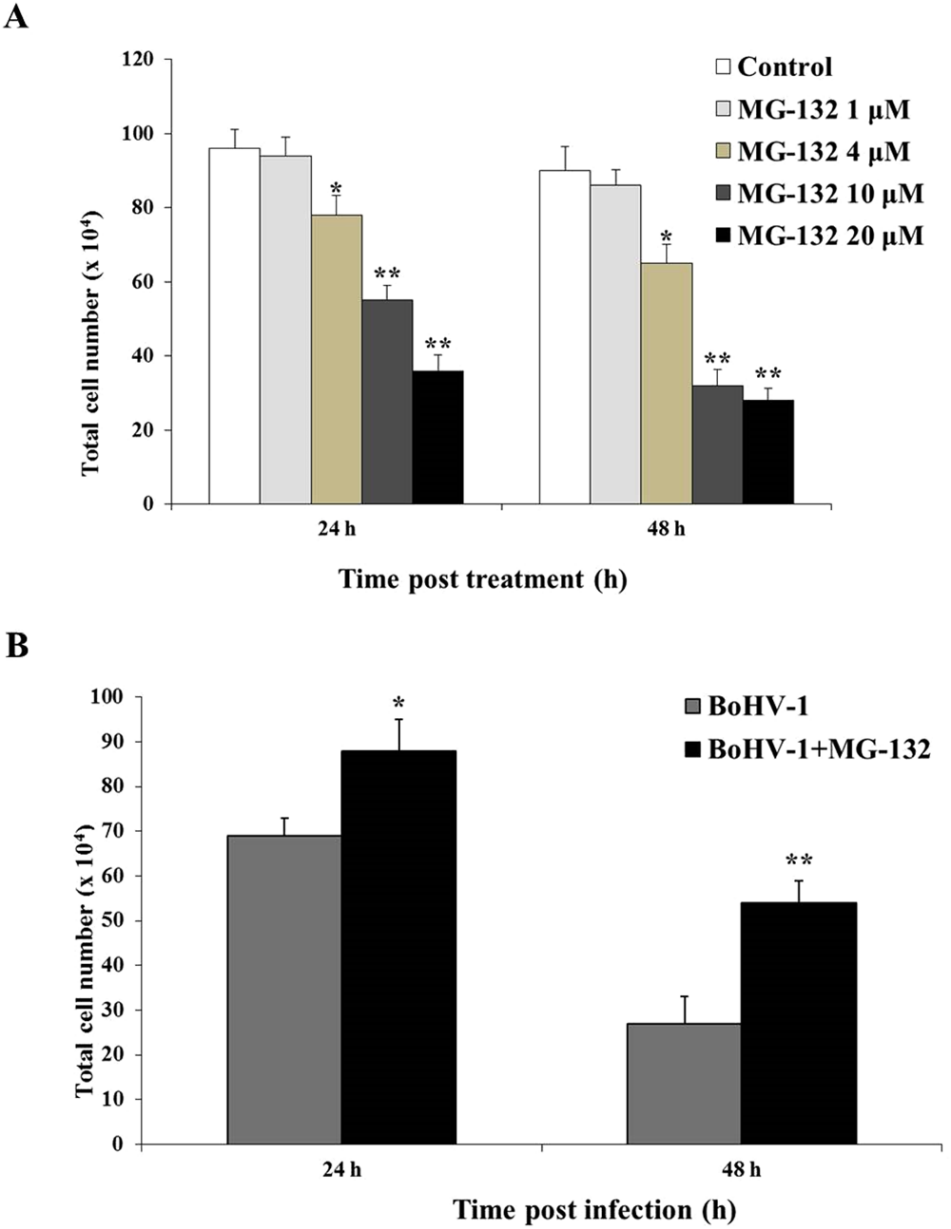
MG-132 decreases cytotoxicity of MDBK during BoHV-1 infection. (**A**) MDBK cells were treated with MG-132 for 4, 24 or 48 h. (**B**) MDBK cells were infected with BoHV-1 alone or in association with MG-132 for 4, 24 or 48 h. At different times of treatment cells were stained with Trypan-blue and scored with a Burker chamber at a light microscope. Data are presented as mean ± S.E.M. of three independent experiments performed in duplicate. Significant differences between infected untreated and infected MG-132-treated groups are indicated by probability P. *P < 0.05 and **P < 0.01.

**Figure 2:**
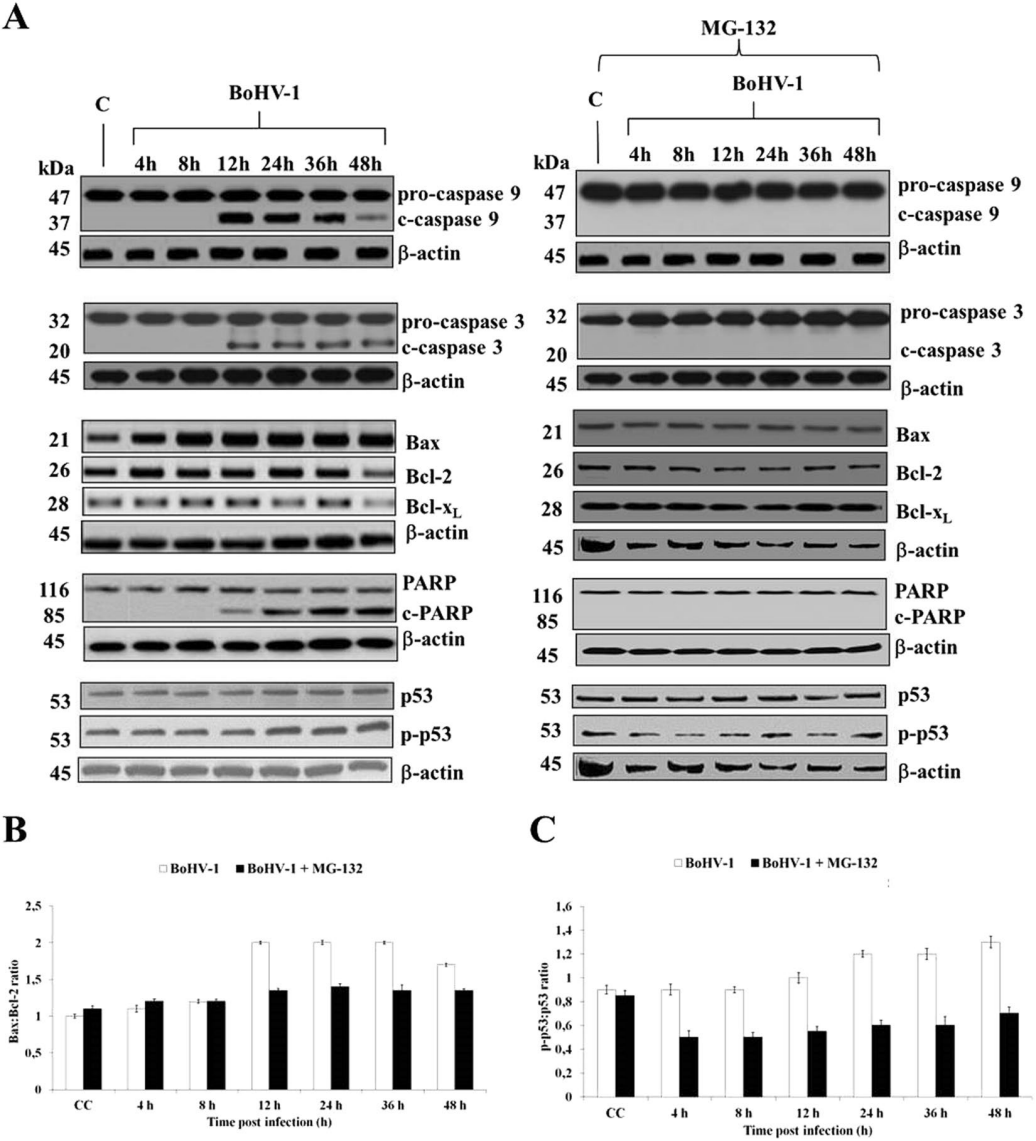
MG-132 inhibits BoHV-1-induced apoptosis in MDBK cells. (**A**) Cell lysate was prepared from mock-infected cells (lanes C), or infected cells, exposed or not to MG-132 and, for the indicated times, Western blot analysis was performed with an antibody which specifically recognized pro-caspase 9, cleaved caspase 9, pro-caspase 3, cleaved caspase 3, Bax, Bcl-2, Bcl-X_L_, PARP, cleaved PARP, p53, p-p53 or β-actin. β-actin was used as an internal loading control. Blots are representative of three separate experiments. (**B**,**C**) Bax/Bcl-2 and p-p53/p53 ratio were obtained by densitometry analysis of the relative blots shown in (**A**). Results are the mean ± S.E.M. of three separate experiments.

The effect of MG-132 on MDBK cell growth was determined by trypan blue exclusion test. We made a dose-response curve at different concentrations (1, 4, 10 and 20 μM). Dose-dependent inhibition of cell growth was observed in MDBK cells with an IC_50_ of approximately 10 μM MG132 for 24 h (Fig. 1A). MG-132 at 1 μM in MDBK cells induced no significant differences in cell viability (P > 0.5) (Figs. 1A, 6A). Moreover, MG-132 did not provoke the activation of caspases 9 and 3 (Fig. 2A). Thus, we choose the concentration of MG-132 of 1 μM to use throughout the study.

In order to evaluate the effect of MG-132 on cell viability during BoHV-1 infection, MDBK cells were infected with BoHV-1 alone or in association with MG-132 (1 μM) and underwent, at different hours p.i., to Trypan Blue exclusion test, as described in Method section. A decrease in viability of BoHV-1 infected cells was observed in a time-dependent manner (Fig. 1B), as previously reported^6,8,9,26,27^. MG-132 induced a significant decrease in cytotoxicity of BoHV-1 infected cells (P < 0.05 and P < 0.01) (Fig. 1B).

### MG-132 inhibits BoHV-1-induced apoptosis in MDBK cells

Analysis of BoHV-1 infected cells exhibited activation of caspases 9 and 3, from 12 h after infection, as well as PARP cleavage (Fig. 2A). BoHV-1 induced phosphorylation of the key tumor suppressor p53 at serine 315 as later as 24 h to the end of infection (Fig. 2A), suggesting phosphorylation levels increased with cell death. Indeed, the infection caused a slight increase of p53 protein levels. The levels of pro-apoptotic member Bax increased as a function of time after infection (Fig. 2), while decreased the levels of anti-apoptotic members such as Bcl-2 and Bcl-X_L_ (Fig. 2).

MG-132 completely inhibited BoHV-1-induced apoptosis, by blocking the activation of initiator caspase 9, as well as, of executioner caspase 3 (Fig. 2). In addition no cleavage of PARP was observed (Fig. 2). These results were accompanied by decrease of both pro-apoptotic and anti-apoptotic members (Fig. 2). From 12 h until the end of the infection, we observed a significant Bax/Bcl-2 ratio which was significantly diminished by MG-132 (Fig. 2A,B) (P < 0.05 and P < 0.01). Our data showed that the levels of both p-p53 and p53 protein levels were decreased by MG-132 (Fig. 2A). Moreover, by measuring the p-p53/p53 ratio, we detected a significant decrease in MG-132 exposed groups, at all times studied (Fig. 2A–C) (P < 0.05 and P < 0.01).

Taken together, our results showed that MG-132 inhibited BoHV-1 induced apoptosis in MDBK cells.

### MG-132 stimulates autophagy in MDBK cells during BoHV-1 infection

**Figure 3:**
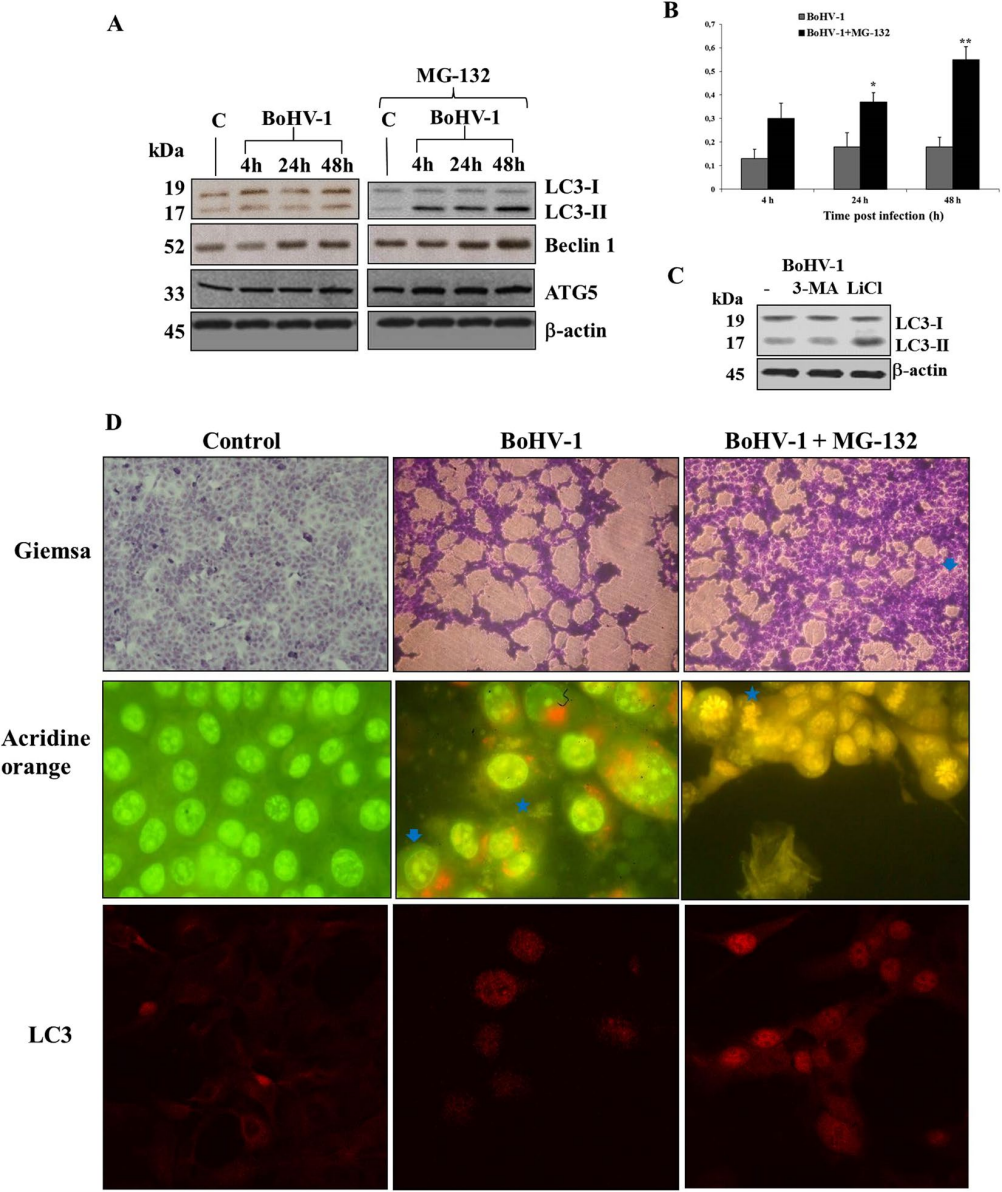
MG-132 stimulates autophagy in MDBK cells during BoHV-1 infection. (**A**) Cell lysate was prepared from mock-infected cells (lanes C), or infected cells, exposed or not to MG-132 and, for the indicated times. Western blot analysis was performed with an antibody which specifically recognized LC3-I, LC3-II, beclin 1, ATG5 or β-actin. Blots are representative of three separate experiments. (**B**) Densitometry analysis of the LC3-II blots shown in (**A**). (**C**) Cell lysate was prepared from infected cells, exposed or not to 3-MA and LiCl for 24 h. Western blot analysis was for LC3-I, LC3-II or β-actin. Blots are representative of three separate experiments. (**D**) Photomicrographs showing morphology of MDBK cells stained with Giemsa. After 48 of infection, compared to the control groups, in MG-132 exposed groups a large number of cells exhibited an increase of characteristic signs of autophagy, such as an elevated degree of vacuolization in the cytoplasm (arrow) which often indicates an increased autophagic flux (magnification × 150); microphotographs of cells staining with acridine orange that revealed the induction of acidic vesicular organelles. Moreover, in infected cells were detected both typical necrotic morphology (star) and apoptotic features (arrow). Whereas, we distinguished only necrotic features in MG-132 infected cells (star). Detection of green and red fluorescence in acridine orange-stained cells was performed using a fluorescence microscope in MDBK control cells, infected and treated or not with MG-132 at 48 h p.i., (magnification × 1000); immunofluorescence for LC3 in infected cells treated or not with MG132 for 48 h, studied as described in Methods Section (magnification × 400).

The influence of MG-132 on autophagic markers was evaluated in MDBK cells during BoHV-1 infection at various times p.i. by Western Blot analysis. LC3 is a recognized marker for mammalian autophagocytosis^28^. LC3 protein is processed during autophagocytosis from an 18 kD protein (LC3-I) to a membrane bound 16 kD protein (LC3-II)^28^. As we showed in Fig. 3A,B, BoHV-1 infection in MDBK cells produced not significant levels of LC3-II. Whereas, MG-132 induced in BoHV-1 infected groups a time-dependent increase in the amount of autophagy by increasing the conversion of the autophagy marker LC3 from LC3-I to LC3-II (Fig. 3A,B) and by enhancing the levels of ATG5, an additional autophagy-related protein markers and of Beclin 1, an autophagy related protein (Fig. 3A)^28^. In addition, we used another inducer of autophagy, like LiCl, which confirmed the activation of autophagy at 24 h p.i. (Fig. 3C). Whereas, 3-MA, an inhibitor of autophagy, at 24 h p.i., induced no significant differences in the level of LC3-II compared to infected control (Fig. 3C).

Furthermore, after Giemsa staining, as shown in Fig. 3D, at 48 h after infection, in MG-132 unexposed groups we detected an increase of intercellular spaces and some morphological alterations that indicated signs of both necrosis and apoptosis. Necrosis is morphologically characterized by swelling of organelles, plasma membrane rupture and subsequent loss of intracellular contents^29^, while apoptosis is accompanied by rounding-up of the cell, retraction of pseudopodes, chromatin condensation, nuclear fragmentation (karyorrhexis), plasma membrane blebbing (but maintenance of its integrity until the final stages of the process)^29^. In MG-132 exposed groups a large number of cells exhibited an increase of characteristic signs of autophagy, such as an elevated degree of vacuolization in the cytoplasm (arrow) (Fig. 3D), which often indicates an increased autophagic flux^28–30^. Furthermore, detection of AVOs, as autophagic signs, was carried out by using lysosomotropic agent acridine orange (Fig. 3D). Autophagy is a process of sequestrating cytoplasmic proteins into the lytic component and to evaluate the AVOs in MG-132 treated cells, we performed the vital staining with acridine orange. At 48 h p.i., in acridine orange-stained cells, the cytoplasm and nucleolus fluoresced bright green and dim red, as resulted both in control and BoHV-1 groups (Fig. 3D), whereas, MG-132 enhanced acidic compartments which fluoresced in bright red (Fig. 3D). BHV-1 infected cells stained by acridine orange displayed both typical necrotic morphology (Fig. 3D - star) and apoptotic features (Fig. 3D - arrow). Whereas, we distinguished only necrotic features in few MG-132 infected cells (Fig. 3D - star).

In addition, after 48 h of infection, MG-132 increased punctate staining for LC3 compared to infected or uninfected controls, as detected by immunofluorescence for LC3, using epitope-specific antibody on paraformaldehyde-fixed cells (Fig. 3D). In particular, when MDBK cells were infected in the presence of MG-132, there was a strong accumulation of LC3-II (Fig. 3D). The amount of LC3 is correlated with the extent of autophagosome formation^28^.

Our results displayed that during BoHV-1 infection MG-132 stimulated MDBK cells to undergo autophagy, in a time-dependent manner.

### MG-132 inhibits protein expression of bICP0 during infection in MDBK cells

**Figure 4:**
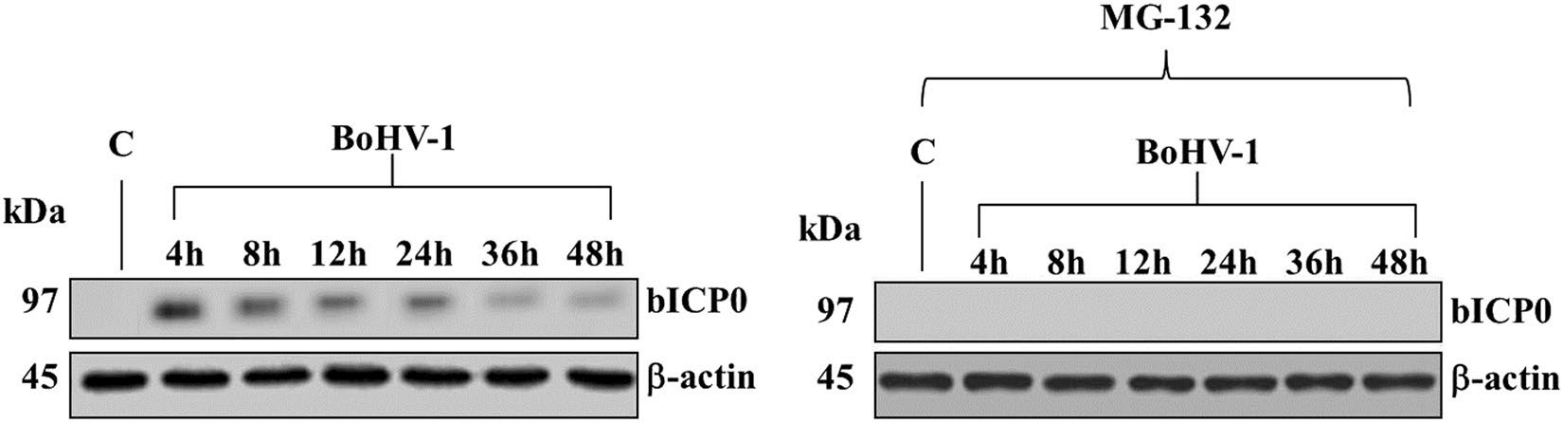
MG-132 inhibits protein expression of bICP0 during infection in MDBK cells. Cell lysate was prepared from mock-infected cells (lanes C), or infected cells, exposed or not to MG-132 and, for the indicated times, Western blot analysis was performed with an antibody which specifically recognized bICP0 or β-actin. β-actin was used as an internal loading control. The molecular weight (in kDa) of protein size standards is shown on the left hand side. Blots are representative of three separate experiments.

Following infection bICP0 has been regularly detected in infected cells since 2 h p.i. until the end of the infection (48 h) (Fig. 4), according to previous studies^4,31^. MG-132 completely inhibited protein expression of bICP0 during BoHV-1 infection in MDBK cells (Fig. 4).

### MG-132 delayed the translocation of NF-κB from cytoplasm to nuclei during infection in MDBK cells

**Figure 5:**
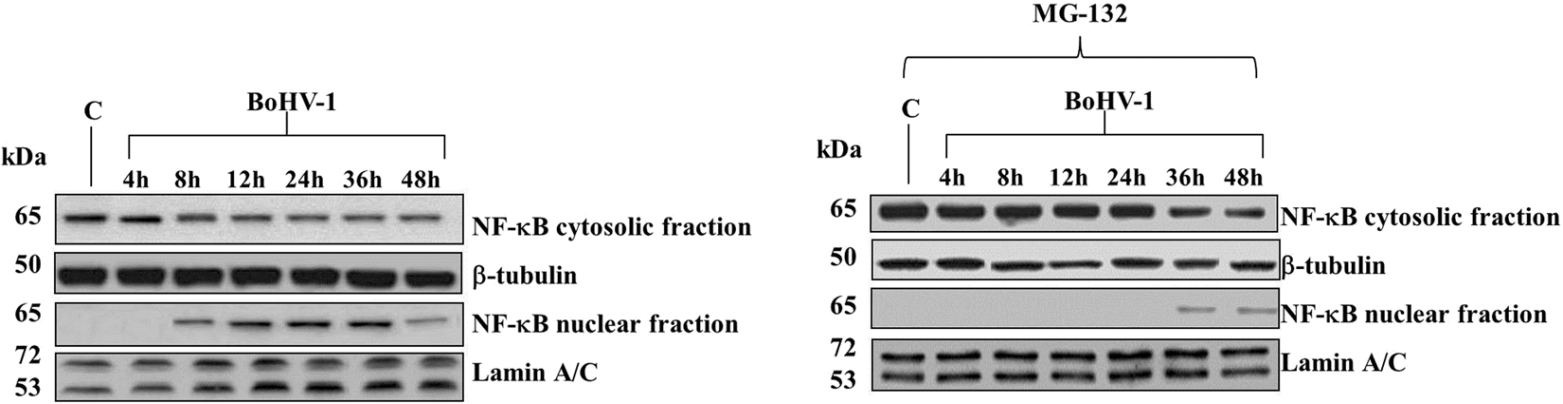
MG-132 delayed the translocation of NF-κB from cytoplasm to nuclei during infection in MDBK cells. Cell lysate was prepared from mock-infected cells (lanes C), or infected cells, exposed or not to MG-132 and, for the indicated times, Western blot analysis was performed with an antibody which specifically recognized NF-κB, β-tubulin or Lamin A/C. β-tubulin and Lamin A/C were used as internal loading controls. The molecular weight (in kDa) of protein size standards is shown on the left hand side. Blots are representative of three separate experiments.

Herein, to verify if MG-132 could determine changes in the NF-κB protein expression during BoHV-1 infection, we measured NF-κB amount using Western blot assay. We detected NF-κB in cytosolic fraction during infection, and in nuclear fraction from 8 h to the end of infection (Fig. 5). The presence of MG-132 significantly delayed its translocation from cytoplasm to nuclei (Fig. 5), where NF-κB was detected in the last time of infection (Fig. 5).

Our results indicated that MG-132 influenced the activation of NF-κB, by delaying its translocation from cytoplasm to the nuclei.

### MG-132 reduces virus production during productive infection in MDBK cells

To verify whether MG-132 exposure could affect the virus production, we analyzed both viral cytopathic effects and virus titration. To this aim MDBK cells were infected with BoHV-1 and exposed or not to MG-132 and processed as reported in Method Section.

**Figure 6:**
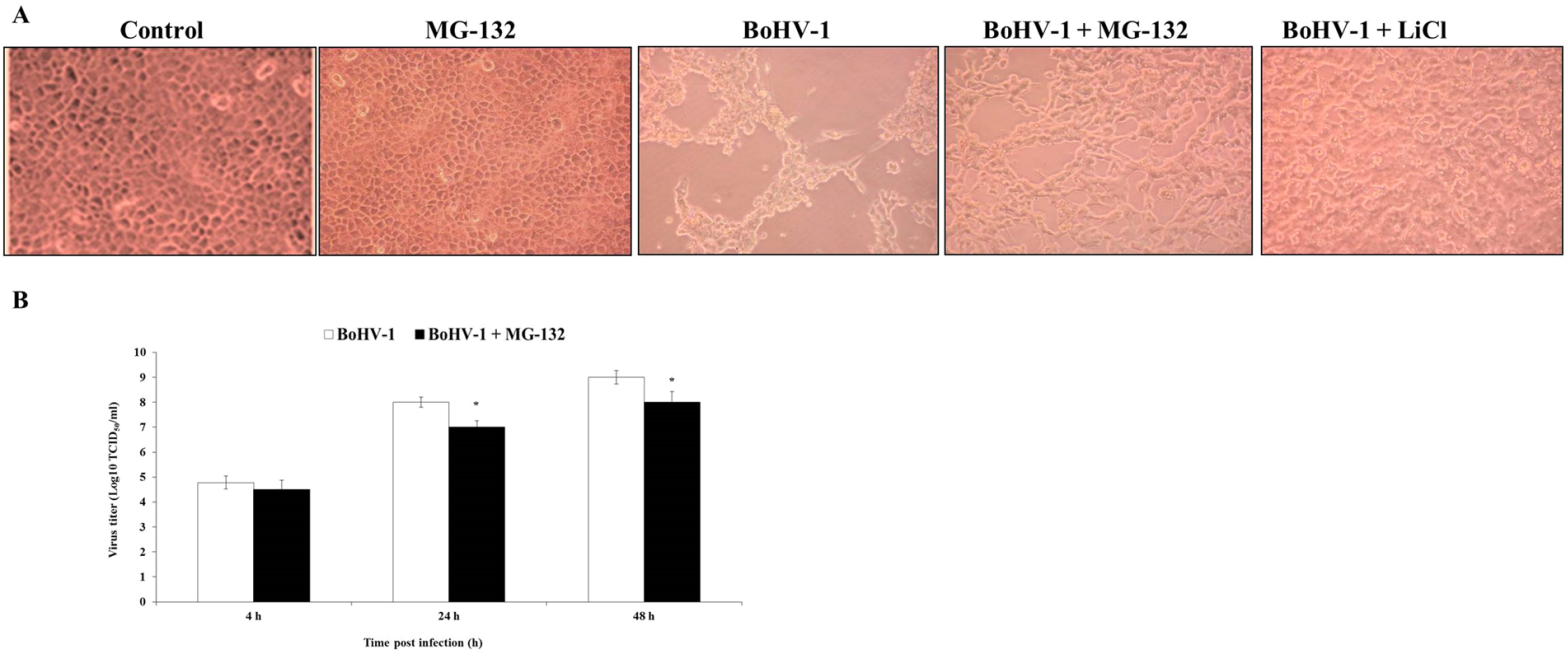
MG-132 reduces virus production during productive infection in MDBK cells. (**A**) Representative microphotographs by phase-contrast light microscopy of MDBK control cells, untreated or treated with MG-132, infected with BoHV-1 and untreated or treated with MG-132 and LiCl at 48 h p.i., showing the cytopathic effects and the morphological changes on cellular monolayers. (**B**) Virus titers, assayed by TCID_50_ method and reported as Log TCID_50_/ml.

The CPE, represented by ample syncytia formation along with elimination of the cellular sheet, was much evident in BoHV-1 unexposed after 48 h p.i. (Fig. 6) and particularly, in MG-132 treated groups it was possible appreciate an intense decrease of CPE (Fig. 6). To confirm that autophagy induction results in loss of BoHV-1 infection, we used lithium, another inducer of autophagy^28^, which similarly to MG132 decreased CPE (Fig. 6).

Virus titers, assayed by TCID_50_ method, confirmed the above data. In fact, from 24 to 48 h p.i., we observed a significant decrease of viral production in MG-132 treated groups (Fig. 6).

Taken together, our data showed that MG-132 reduced virus production.

## Discussion

The proteasome system is the major pathway of intracellular protein degradation in eukaryotes. Several studies showed that some viruses can use the host ubiquitin-proteasome pathway for their own needs. For example, functional proteasomes facilitate HSV-1 entry at a post-penetration stage^25^. Indeed, proteasome inhibitors impair incoming HSV-1 capsid transport to the nuclear periphery but do not affect cell attachment, penetration, or membrane fusion^25^. To date, whether the ubiquitin-proteasome pathway mediated BoHV-1 replication remains largely unknown. Thus, the aim of this study was to explore the effect of MG-132, a proteasome inhibitor, on the BoHV-1 replication in MDBK cells. Moreover, we analyzed the expression of viral and cellular proteins and their role during viral infection.

MG-132 induced a time-dependent decrease in cytotoxicity of infected cells and inhibited BoHV-1-induced apoptosis in MDBK cells (Fig. 2). In particular, analysis of apoptotic pathway showed the activation of both examined caspases and the PARP cleavage (Fig. 3), according to previous studies^4,6,8–11^. MG-132 completely inhibited BoHV-1-induced apoptosis, by blocking the activation of initiator caspase 9, as well as, of executioner caspase 3, and no cleavage of PARP was observed (Fig. 3). The levels of pro-apoptotic member Bax increased as a function of time after infection, while decreased the levels of anti-apoptotic members such as Bcl-2 and Bcl-XL (Fig. 3)^6,8^. These results were accompanied by a decrease of both pro-apoptotic and anti-apoptotic members. In particular, we detected that Bax/Bcl-2 ratio was significantly decreased by MG-132 (Fig. 3). Key tumor suppressor p53 typically contributes to BoHV-1-induced apoptosis^6^ and Ser315 locates within the nuclear localization signal (aminoacids 305-322) of the C-terminal region of p53. Ser315 is phosphorylated by kinases involved in cell cycle progression, such as cyclin-dependent kinase cdk9^32^. It has been shown that Ser315 phosphorylation is required for the E2F family to enhance p53-mediated transcription and apoptosis^33^. Thus, we choose to study Ser315 phosphorylation and we found that BoHV-1 induced p53 phosphorylation at serine 315 as later as 24 h to the end of infection (Fig. 3), suggesting that phosphorylation levels increased with cell death, as we previously detected in the same model of cell line^34^. Indeed, in the absence of MG-132, the infection slightly induced p53 protein levels^6^. Here, both p-p53 and p53 protein levels were decreased by MG-132 (Fig. 2A). Moreover, by measuring the p-p53/p53 ratio, we detected a significant decrease in MG-132 exposed groups, at all times studied (Fig. 2A-C). Taken together, those results confirmed that, in bovine cells infected with BoHV-1, MG-132 did not induce significant apoptosis, as in cells infected with HSV-1^35^.

Infection of MDBK cells with the bICP0 null mutant, a BoHV-1 recombinant virus, leads to an accumulation of autophagosomes that are not detected following infection with BoHV-1^7^. Herein, we used an inhibitor of autophagy, like 3-MA (Fig. 3)^28^. Our results provided evidence that a small amount of autophagy takes place during BoHV-1 infection in MDBK. It is well established that necrosis is the predominant type of cell death in infected cells. Apoptotic cell death only occurs near the end of productive infection in MDBK cells^6–8^. Following infection of MDBK cells, biochemical and morphological results showed that MG-132 induced autophagy (Fig. 3), as well as strongly inhibited the activation of bICP0 (Fig. 4). Moreover, MG-132, as LiCl (Fig. 3B), caused a significant suppression of viral yields in bovine cells (Fig. 6). It is known that bICP0 acts as an ubiquitin ligases and its activating effect was attenuated by MG-132^17^. Interestingly, bICP0 null mutant, a virus generated by homologous recombination, stimulates the formation of cytoplasmic vesicles similar to autophagosomes, suggesting that bICP0 null mutant induced autophagy in MDBK cells but not in rabbit skin cells^7^. Intriguingly, MG-132 infected cells, stained by acridine orange, appeared with rounded nuclei and elongated fusiform morphology (Fig. 3), similar to cultures infected with the bICP0 null mutant^36^. Moreover, bICP0 null mutant induces low levels of apoptosis^7^ and does not produce plaques by growing weakly in bovine cells^36^. In fact, it has been demonstrated that infection of bovine cells with bICP0 null mutant resulted in at least 100-fold lower virus titres, indicating that bICP0 protein expression is important, but not required, for virus production^36^.

Entry of BoHV-1 into cells, such as HSV-1, is a multistep process which involves the host cell machinery. Herein, MG-132 was added to cells at the time of infection and, as above reported, it has been known for some time that MG-132 treatment inhibits transport of the HSV-1 capsid to the nucleus^25^. Inhibition of BoHV-1 infection at such a very early stage (prior to delivery of the genome to the nucleus) is suggested by the complete lack of expression of the viral immediate early activator bICP0 at any time point in the presence of MG-132 (Fig. 4). BoHV-1 strains null for ICP0 are severely attenuated for lytic replication^36^ and thus, in the absence of detectable bICP0 protein, and with no data showing expression of downstream viral genes, it is unclear to what extent MG-132 is exerting any of its effects on BoHV-1 replication, as opposed to initial entry. Reduced initial infection would also explain reduced cytopathic effect, and reduced final titer (Fig. 6), seen in the presence of MG-132. It has been proposed that proteasome-mediated degradation of a virion or host protein is regulated by ICP0 to allow efficient delivery of entering HSV-1 capsids to the nuclear periphery^37^. But, ICP0-deficient virions are not blocked by proteasomal inhibitors and enter cells via a proteasome-independent mechanism^37^. Thus, we hypothesize that BoHV-1, like HSV-1, may enter cells in an ubiquitin independent manner, as HSV-1 successfully entered cells in the absence of a functional host ubiquitin-activating enzyme^25^. We also suppose that MG-132 may have a reversible inhibitory effect on BoHV-1 entry, such as occurs in HSV-1 infection^25^. Finally, inactivation of the ubiquitin-proteasome pathway inhibits viral replication in a manner that closely follows multiplicity and cell-type dependence of the requirement for ICP0^38^, thus to elucidate the role that MG-132 plays with respect to BoHV-1 infection, it will be necessary to realize further studies.

Signaling events leading to NF-κB activation constitute a major antiviral immune pathway. Particularly, activation of NF-κB transcription pathway is crucial for the immediate early step of immune activation. To replicate and persist within their hosts, viruses have evolved different strategies to evade cellular NF-κB immune signaling cascades for their benefit^16^. BoHV-1 shares certain biological properties with HSV-1^1,2^. And in HSV-1 cellular proteasome activity inhibitors prevent virus induced NF-κB activation in the early phase of infection and decreases immediate early as well as late protein expressions, and suppresses viral replication^18^. Our results suggest that, in the presence of MG-132, the inhibition of bICP0 could significantly delayed the activation of NF-κB (Fig. 5).

Interestingly, MG-132 impaired the entry of Japanese encephalitis virus (JEV) and its initial translation. The internalized JEV viral particles were misdirected to the lysosomes, resulting in non-productive entry^39^. This paper proposed a dramatically different role for MG-132 than those proposed by other authors and here. But JEV is a positive-stranded enveloped RNA virus that belongs to the family Flaviviridae whereas, it has been reported that double-stranded-DNA-mediated NF-κB activation is critical for host antiviral responses. Indeed, the HSV-1 ubiquitin-specific protease (UL36USP), which is a deubiquitinase (DUB), antagonizes NF-κB activation, depending on its DUB activity^40^. The N terminus of the HSV-1 UL36 gene-encoded protein contains the DUB domain and is conserved across all herpesviruses. UL36USP abrogates NF-κB activation by cleaving polyubiquitin chains from IκBα and therefore restricts proteasome-dependent degradation of IκBα and DUB activity is indispensable for this process^40^.

**Figure 7:**
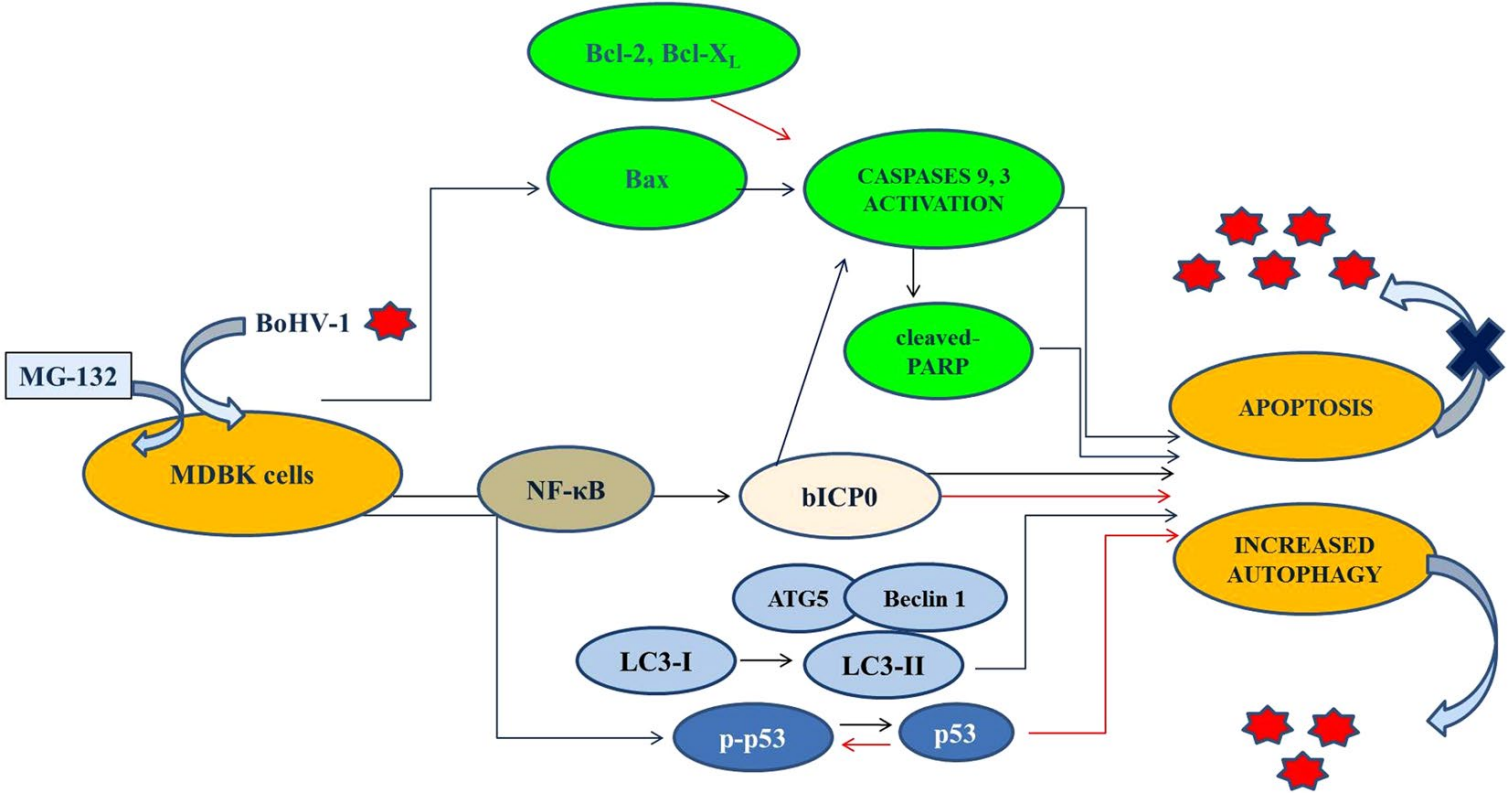
Schematic diagram illustrating the hypothesized mechanisms as to how MG-132 exerts its effects on the BoHV-1 replication, resulting in stimulated autophagy and decreased virus replication in MDBK cells.

An important virulence mechanism of HSV-1 is the control of autophagy. In fact, HSV-1 inhibits autophagic responses through binding of the viral protein ICP34.5 to the host protein Beclin1^41^. Recently, Yakoub and Shukla argued that MG-132 stimulates autophagy in HSV-1 infection and suppresses infection in various cell types^35^ (2015). Emerging evidence suggests that during infection, autophagy may act as an antiviral mechanism, by decreasing the replication, or enhancing the degradation of many viruses^42^. Our findings indicate that the efficient virus release provoked by BoHV-1 may be lowered by proteasome inhibitor (See Diagram in Fig. 7). Furthermore, in BoHV-1 infection MG-132 might stimulate autophagy, a highly conserved cellular degradative pathway, and significantly limit infection in bovine cells, without affecting cell viability, according to the theory that autophagy induction may prevent cell death. Indeed, autophagic activity of a cell was shown to be inversely proportional to the probability of its death^43,44^.

In many European countries BoHV-1 is widespread and IBR eradication still represents a goal^45–47^, as the virus is responsible for significant losses incurred by disease and trading restriction in the cattle industry^2^. Thus, we suppose that the activation of autophagy may provide an antiviral defense mechanism in infections caused by the BoHV-1.

## Methods

### Cell cultures and virus infection

MDBK cells (American Type Culture Collection, CCL22) were cultured in Dulbecco’s modified Eagle’s minimal essential medium (DMEM), supplemented with 2% foetal calf serum (FCS), 1% L-glutamine, 1% penicillin/streptomycin, 0.2% sodium pyruvate and 0.1% tylosin, a macrolide-class antibiotic. Cells were maintained in an incubator at 37 °C (in 5% CO_2_/95% air). This cell line was maintained free of mycoplasma and of bovine viral diarrhoea virus as described^9^. The BoHV-1 Cooper strain was used throughout the study. Virus stocks were routinely grown on MDBK cells and were also used for determination of virus titers^26,27^. Proteasome inhibitor carbobenzoxy-L-leucyl-L-leucyl-L-leucinal (MG-132) (Calbiochem, 474790) was dissolved in dimethyl sulfoxide and added to the media to a final concentration of 1 μM. In addition, we used both an inducer and an inhibitor of autophagy, 10 mM lithium chloride (LiCl) and 10 mM 3-methyladenine (3-MA), respectively (all from Sigma-Aldrich). All other chemicals were of the highest commercially available purity.

MDBK cells, at confluency, were washed with DMEM and then infected or not with BoHV-1, at multiplicity of infection (MOI) of 5, at the same time, in the presence or absence of MG-132. After 1 hour of adsorption at 37 °C, the cells were incubated for indicated times post infection (p.i.) and then processed. The virus was present in the culture media throughout the course of the experiment.

### Cell viability

Cell viability was assessed by Trypan blue (TB) exclusion test, as we described^4,8^. Briefly, cells were collected by trypsinization, at the indicated times after infection, and an aliquot of the cell suspension was mixed with an equal volume of 0.2% Trypan-blue (Sigma) in 1 × phosphate-buffered saline (PBS). After 10 min, cells were counted using a Burker chamber under a phase contrast microscope. Cell viability was calculated as percentage of live cells over the total cells number, and results are the mean ± S.E.M. of four independent experiments performed in duplicate.

### Examination of cell morphology

Cell morphology was examined by light microscopy following Giemsa staining^34^. Briefly, monolayers of MDBK cells (10^5^ cells per well), in 16-well culture chambers, were infected or not with BoHV-1, at MOI of 5, in the presence or absence of MG-132, and incubated at 37 °C. After 4, 24 and 48 h p.i., cells were washed twice with PBS. Cells were fixed with 95% ethanol, drained and dried. Afterward, cells were stained with a 5% Giemsa solution (Merck, 109203). After 30 min cells were rinsed with tap water and H_2_0. The slides were mounted in Entellan (Merck, 100869) and coverslipped. Light microscopic studies and photomicrographs observations were carried out. Non-apoptotic cell death was identified by the criteria previously described^30,48,49^.

Moreover, we detected acidic vesicular organelles (AVOs) with acridine orange as previously described^34,50^. Autophagy is the process of sequestrating cytoplasmic proteins into the lytic component and it is characterized by the formation and promotion of AVOs. In order to detect the AVO in cells, we carried out the vital staining with acridine orange. In acridine orange-stained cells, the cytoplasm and nucleolus fluoresce bright green and dim red, whereas acidic compartments fluoresce bright red^51,52^. The intensity of the red fluorescence is proportional to the degree of acidity of the cellular acidic compartment. Monolayers of MDBK cells grown on glass coverslip, at confluency, were washed with DMEM and were infected or not with BoHV-1, at MOI of 5, in the presence or absence of MG-132, and incubated at 37 °C. After 4, 24 and 48 h p.i., cells were washed with PBS and stained with acridine orange (Sigma, A6014) at a final concentration of 1 μg/ml for 15 minutes. Stained cells were placed on a microscope slide and observed under UV with a fluorescence microscope (Nikon).

### Immunofluorescence and confocal analysis

Monolayers of MDBK cells grown on glass coverslip, at confluency, were washed with DMEM and were infected or not with BoHV-1, at MOI of 5, in the presence or absence of MG-132, and incubated at 37 °C. At 4, 24 and 48 h p.i., cells were washed with PBS and then fixed with paraformaldehyde (4% w:v). After rinsing in PBS, the cells were permeabilized with 0.1% Triton X-100 in PBS for 1 min and blocked in 4% BSA in PBS for 30 min. This was followed by incubation in rabbit polyclonal microtubule-associated protein light chain 3 (LC3) antibody (1:200) (Novus Biologicals, NB100-2220), for 24 h at 4 °C in a humidified chamber. After 3 washes in PBS, the cells were incubated in anti-rabbit IgG conjiugated to Cy3 (1:150) (Sigma, C2306) for 1 h at room temperature. Then, cells were rinsed in PBS, coverslipped and examined with a confocal microscope (Zeiss).

### Isolation of nuclear and cytosolic fractions

Isolation of nuclear and cytosolic fractions was carried out as previously described^4^. MDBK cells were harvested and washed twice with ice cold PBS, then centrifuged at 1,200 rpm for 7 min at 4 °C. Pellets were re-suspended in 0.33 M sucrose, 10 mM Hepes (pH 7.4), 1 mM MgCl2, 0.1% Triton X-100 in 5:1 v/v (ice cold and in the presence of protease inhibitors). After 15 min on ice, aggregates were broken up gently with thin glass rod and centrifuged at 3,000 rpm for 5 min at 4 °C. The supernatants representing the cytosolic fractions and the pellets containing a pure preparation of nuclei without the nuclear membrane were obtained and stored at −70 °C. Nuclear pellets were gently re-suspended in ice cold 0.45 M NaCl, 10 mM Hepes pH 7.4 (in the presence of protease inhibitors). The suspension was incubated on ice for 15 min. After centrifugation at 14,000 rpm for 5 min at 4 °C, supernatant representing the nuclear extract was collected and then Western blot analysis of nuclear and cytosolic fractions were performed to detect NF-κB.

### Protein extraction and Western blot analysis

Protein extraction and Western blot analysis were carried out as we previously described^4,8,9,34^. The following antibodies, dissolved in 5% bovine serum albumin-TBST, were used: anti-caspase-3 PAb (1:1000) (Santa Cruz Biotechnology, sc-1225), anti-caspase-9 PAb (1:500) (Acris Antibodies, Inc., AP00004PU-N), anti-p53 MAb (1:1000) (Santa Cruz Biotechnology, sc-99), anti-phospho-p53 PAb (Ser315) (1:1000) (Cell Signaling, 2528), anti-PARP Mab (1:5000) (Biomol, BML-SA250-0050), anti-Bax Pab (1:1000) (Abcam, ab7977), anti-Bcl-2 Pab (1:1000) (Abcam, ab7973), anti-Bcl-X_L_ Pab (1:1000) (Abcam, ab2568), polyclonal rabbit anti-bICP0 (a.a. 663–676) serum (1:800), kindly provided by Prof. M. Schwyzer (University of Zurich, Switzerland)^3^, anti-LC3 PAb (Novus Biologicals, NB100-2220) (1:1500), anti-ATG-5 (Novus Biologicals, NB110-53818) (1:500), anti-Beclin 1 PAb (Novus Biologicals, NBP1-00088) (1:1000), anti-NF-κB Mab (1:1500) (Imgenex, IMG150B), antin-Lamin A/C MAb (Sigma, SAB4200236), anti-β-tubulin PAb (Abcam, ab6046) and anti-β-actin MAb (Sigma, A5316) (1:7500). The images of Western immunoblot specific bands on X-ray films were imported into a computer by a scanner and captured as digital TIFF images. The results were plotted in a graph after densitometry analysis of the obtained blots. Moreover, both Bax/Bcl-2 and p-p53/p53 ratio in infected groups, in the presence or in absence of MG-132, were obtained by densitometric evaluation.

### Virus production

MDBK cells, at confluence, were infected with BoHV-1 at MOI 5, in the presence or not of MG-132 and LiCl, as above reported. For the virus production assay, we analyzed both viral cytopathic effects (CPE) and virus titration. At 24 or 48 h p.i., all groups were observed under light microscope to evaluate CPE, represented by ample syncytia formation along with elimination of the cellular sheets. For virus titration, at 4, 24 or 48 h p.i., cell extracts, obtained by three cycles of freezing and thawing, were collected and stored in aliquots at −80 °C. Virus titers were assayed by TCID_50_ method according to Reed and Muench (1938)^53^, as previously described^26,27^.

### Statistical analysis

Data are presented as mean ± S.E.M. One-way ANOVA with Tukey’s post-test was performed using GraphPad InStat Version 3.00 for Windows 95 (GraphPad Software, San Diego, CA). *P* value < 0.05 was considered statistically significant.
